# The Strange Case of the Nuragic Offerers Bronze Statuettes: A Multi-Analytical Study

**DOI:** 10.3390/ma15124174

**Published:** 2022-06-13

**Authors:** Antonio Brunetti, Marta Porcaro, Sergio Lins, Francesco di Gennaro, Rosario Maria Anzalone, Mario Mineo, Anna Depalmas

**Affiliations:** 1 Dipartimento di Scienze Biomediche, Università degli Studi di Sassari, 07100 Sassari, Italy; 2 Dipartimento di Chimica e Farmacia, Università degli Studi di Sassari, 07100 Sassari, Italy; mporcaro@uniss.it; 3 Dipartimento di Scienze di Base e Applicate per L’ingegneria, Università degli Studi di Roma “La Sapienza”, 00161 Rome, Italy; sergio.lins@roma3.infn.it; 4 Ex– Museo Preistorico Etnografico Luigi Pigorini, 00144 Rome, Italy; francescodig@gmail.com; 5 Musei Reali di Torino, 10122 Torino, Italy; rosariomaria.anzalone@beniculturali.it; 6 Museo Preistorico Etnografico Luigi Pigorini, 00144 Rome, Italy; mario.mineo@beniculturali.it; 7 Dipartimento di Scienze Umanistiche e Sociali, Università degli Studi di Sassari, 07100 Sassari, Italy; depalmas@uniss.it

**Keywords:** nuragic bronze, NDT, XRF, cultural heritage

## Abstract

The Nuragic civilization (Sardinia, Italy, XVIII–VIII B.C) developed a flourishing bronze metallurgy with strong connections with other civilizations from the Mediterranean basin. Within the large bronze production, there are some peculiar representations of human figures, known in the archaeological environment of Sardinia as bronzetti, depicting warriors, priests, and offerers. In this paper, an interesting couple of Nuragic statuettes representing offerers, one from the Pigorini Museum in Rome and another from the Musei Reali in Turin, were analyzed. They have been investigated with X-ray fluorescence integrated with Monte Carlo simulations (XRF-MC). The combined methodology provides more accurate results, ranging from the structural characterization to the identification of the corrosion layers to the estimation of the composition of the alloy of the artifact. One of the most striking results regards the heads of the offerers: both heads are covered with a thick iron-based layer, even though the whole artifacts are made of a copper alloy. To understand the reason behind this peculiar corrosion patina, several hypotheses have been considered, including the possibility that these iron mineralizations are the consequence of an ancient superficial treatment, intending to confer a chromatic effect on the figurine’s head.

## 1. Introduction

The Nuragic civilization is born on the island of Sardinia, Italy, around 1700 B.C. It developed a magnificent and original architecture testified by the truncated conical tower buildings called “nuraghe” (to this day, about eight thousand nuraghi have been discovered). Furthermore, the Nuragic people have also developed a remarkable metallurgic production, essentially of bronzes [1,2]; reaching its full development by the end of the Bronze Age and the Early Iron Age. Such craftsmanship comprises not only metallic tools, such as axes or swords, or the small ship models known as navicelle, but many anthropomorphic figurines representing warriors, priests, and offerers as well. Unfortunately, most of these bronze objects were collected through illegal excavations, making it very difficult to establish their archaeological context and also the establishment of their provenance. It also hinders the study of the chronology and geographical evolution of the Nuragic people’s craftsmanship.

The scarce available information from such objects comes from the object’s alloy composition and morphological structure. For these reasons, it is primordial to extract all information possible from the composition and microstructure of the alloy. Several non-systematic studies have been performed both with destructive and non-destructive approaches [2,3,4,5]. However, only a few general conclusions were obtained.

**Figure 1:**
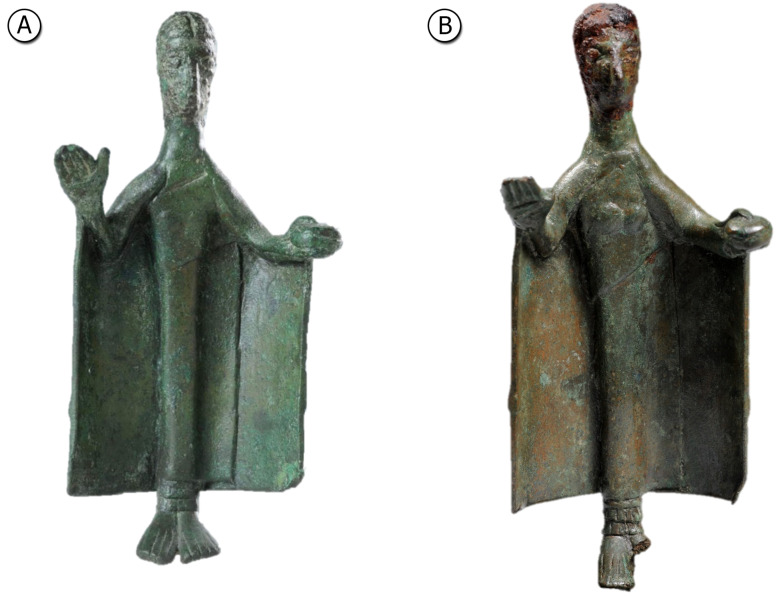
Bronze statuettes: (**A**) Pigorini Museum (#26065) and (**B**) Musei Reali Museum (#783).

In this framework, considering the availability of new analytical techniques, such as neutron diffraction (ND) and neutron imaging (NI), or X-ray fluorescence (XRF) integrated with Monte Carlo simulations, a few years ago some of us have started a new systematic campaign of investigations on Nuragic artifacts [6,7,8]. In the last year, a new group of samples, coming from the Museo Nazionale Preistorico Etnografico “Luigi Pigorini” in Rome and Musei Reali in Turin, have been analyzed. This group of samples includes two small statuettes representing each a female figure (Figure 1). The women are presented in a solemn posture with an offer in the left hand and the right hand in a greeting attitude. They have a long mantle and appear to be a representation of a high-rank person, perhaps an offerer. Although at a first glance they appear to be made of a typical Nuragic bronze, XRF measurements have revealed a peculiarity: both heads are covered with a thick iron-based layer. The remaining parts of the body are covered with a corrosion patina. Both types of corrosion (from iron and bronze), are due to the interaction of the original material/alloy with the burial environment. Different sediments composition can produce different types of corrosion.

## 2. Materials and Methods

In the present investigation, two main techniques have been used and combined: X-ray fluorescence (XRF) and Monte Carlo simulations (MCS). They are discussed in the following paragraphs independently.

### 2.1. X-ray Fluorescence

XRF instrumentation is relatively inexpensive and portable, allowing researchers to perform measurements inside museums and galleries. Furthermore, it is a non-destructive technique, making it very appealing to the Cultural Heritage field, now being a staple in almost every investigation.

The XRF technique bases itself on the interaction of X-ray photons with the object under investigation. This interaction produces an X-ray emission spectrum: a histogram of the number of photons emitted by the sample (secondary photons) and their energy. This spectrum is roughly composed of a background signal on top of which there are a series of peaks. The energy of each peak can be directly related to the presence of a specific chemical element, and its integral can be related to that element’s concentration. However, the peak area depends also on multiple interactions and more in general from the matrix composition.

XRF is essentially a surface technique where only up to the first 100–200 μm are “seen”, depending on the composition of the sample. Usually, on polished surfaces, this is not a limiting factor, but in the case of ancient metallic samples, the surface composition can be altered by corrosion phenomena as well as by restoration processes [9]. Corrosion plays an important role in the case of bronze alloys. It is possible to observe superficial enrichment both of tin and lead [10,11].

A simple XRF measurement can often lead to a wrong estimation of the real bulk alloy composition. To solve this problem, in the last years, a different approach has been used, based on the integration of XRF measurements with Monte Carlo simulations.

### 2.2. Monte Carlo Simulations

MCS are probabilistic statistical mechanical techniques, which are used when a mathematical problem contains several variables that cannot be approximated using traditional analytical techniques [12]. In our case, a custom fast Monte Carlo code called XRMC [13], which exploits the Xraylib atomic database, was used [14,15]. XRMC can produce spectra with the same statistical quality as the measured ones in less than one minute. This approach allows modeling the sample as a multilayer structure where the inner layer, in this case representing the bulk of the alloy, is covered by one or more corrosion layers (the mineralization patina).

Using multi-layered structures it is possible to model the surface enrichment of tin as well as the presence of a protective layer added in past restorations/interventions, otherwise not directly detectable by simple XRF measurements. This approach has been applied in the analyses of many bronzes [16,17,18,19]. A recent article further describes the method applications to a tin enriched sample under a corrosion layer, where a performance similar to that of destructive techniques such as Neutron Activation Analysis (NAA) and Atomic Absorption Spectroscopy (AAS) was achieved [5]. Lastly, the method can simulate more complex structures, such as gradient-like ones.

### 2.3. Quantification Protocol

The protocol for the XRF-MC estimation can be subdivided into two parts. The first part regards the modeling of the experimental setup. It is fundamental to obtain a high-quality reproduction of the X-ray beam under the same conditions as in the real experiment. Here, the emitted spectrum from the X-ray tube is measured by placing the Rh-anode X-ray tube in front of the detector, about 1 m far, to minimize dead-time. Then, the measured spectrum is corrected for the attenuation of air and the response/attenuation of the detector. After this step, the detector is finally modeled. Lastly, the geometry of the setup is replicated inside the Monte Carlo code. These preliminary steps are independent of the sample and can be used theoretically for any measurement with the same setup.

The sample must then be modeled both in composition and structure. The first hypothesis can be obtained by observing characteristics of the surface (morphology, color, etc.), and eventually by the presence of a protective layer (bright translucent appearance or the knowledge about a previous restoration intervention).

By means of a simple XRF measurement, it is possible to identify the chemical elements in the investigated area. The sample composition and structure are the only part of the entire protocol that will be tuned along with the simulations. A simulation is performed, then the spectrum obtained is compared to the measured one, and if any differences are observed, the sample model is updated and a new simulation is started. These three steps are repeated until an almost-perfect match between the real spectrum and the fit is obtained.

However, this estimation requires setting the values for a large number of parameters, such as the number of layers, their compositions, and their thicknesses; driving the problem to a so-called ill-posed problem where, at first, more than one “good” solution can be obtained. For this reason, a good approach is to test several models until the best solution inside a set of good solutions is obtained, by applying a chi-squared test and not only a visual comparison.

The XRF spectrometer is composed of an Rh-anode X-ray tube working at 40 kV, between 5–15 μA, 1 mm-wide collimated, and of an SDD detector without any collimation. The geometry can be changed according to the shape of the sample and its accessibility. Usually, the detector is placed orthogonal to the sample surface with the detector at 45 degrees.

## 3. Results and Discussion

The bulk composition estimations obtained with the XRF-MC method for the two statuettes are summarized in Table 1. The error in the estimated composition is around 5% of the value reported. This error comes from the errors in the atomic parameters involved in the quantification.

The alloy composition of the statuettes is quite similar. Copper concentration varies from 86.8% to 91%, tin from 7.1% to 8.6%, and lead from 1.7% to 4.5%. Except for the lead contents, the other variations are within the experimental errors and intrinsic alloy variations caused by the melting process. From a more general perspective, both artifacts are made of a ternary alloy composed of Cu, Sn, and Pb. The presence of Pb, about 2 wt%, suggests an intentional addition of this element, knowingly used to improve the fluidity and castability of the molten metal [20,21,22].

For both artifacts, a bronze alloy with low tin content was used, favoring cold working annealing [20]. The addition of tin, in concentrations lower than 10 wt%, allows to increase the mechanical characteristics of the bronze and avoids the formation of the eutectoid phase, which makes the alloy more brittle and hard and, thus, more prone to fracture [21]. The ternary alloy composed of Cu, Sn, and Pb was common in the Late Bronze Age. The presence of Pb, in quantities greater than 2 wt%, usually indicates an intentional addition. Lead, due to its slower solidification (its melting point is around 327.5 °C), improves the fluidity and castability of the melt [22,23]. Furthermore, Pb, being cheaper than Cu and Sn, was sometimes also used as a “filler”, even though a high concentration could make it difficult to process the alloy since Pb is immiscible in Cu-Sn [24].

The #26065 statuette has been examined at the spots indicated in Table 2, upper block. For each analyzed spot, the surface of the sample was modeled testing several types of structure, where a three-layer model yielded the best fit. This model is formed by a protective layer placed by the restorer (probably a Paraloid-like film), a corrosion layer, and lastly the bulk alloy. The presence of Sn enrichment layers, due to the decuprification phenomena [10] was also tested, but without any result. The latter hypothesis is consistent with the level of concentration of Sn estimated. Enrichment layers have been detected by Monte Carlo simulations in other bronze analyses only where Sn concentrations were 9–10 wt% or higher [5].

**Figure 2:**
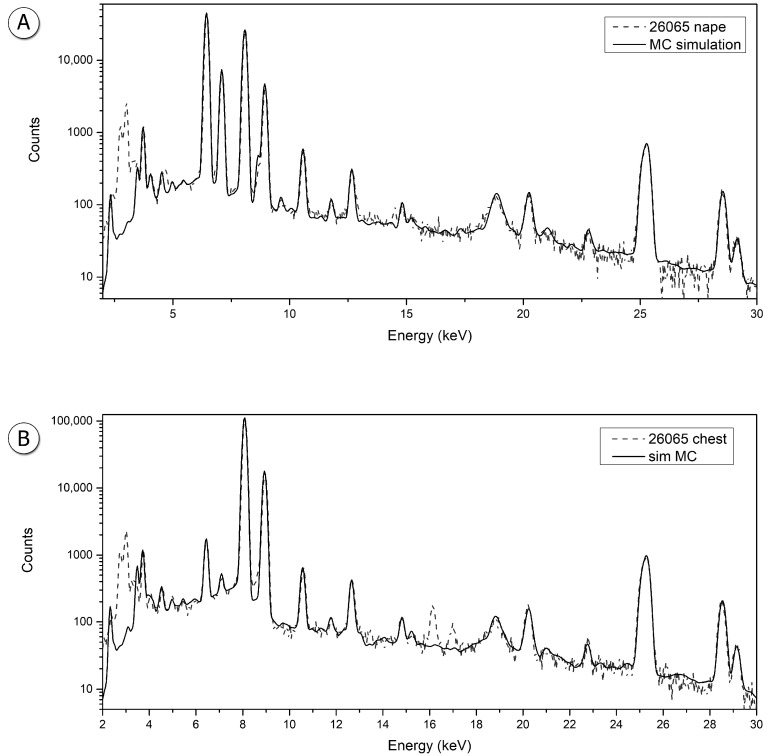
Simulated spectra superimposed to the measured one: sample #26065, (**A**) nape (**B**) and chest.

Statuette #26065 shows a high presence of Fe all around the head and neck, up to a depth of 80 μm under the surface itself, at variable concentrations, while at greater depths only a bronze alloy is detected. The remaining parts of the body are made of a bronze alloy covered by a corrosion patina. However, the Fe-rich layers show different concentrations of Sn with respect to the patina and the lack of a tin enriched layer, which can be due to the “protective” effect of the Fe-based layer, partially stopping the decuprification phenomenon of the bronze alloy. The simulated XRF spectra superimposed to the measured ones for both the head and chest are shown in Figure 2.

**Figure 3:**
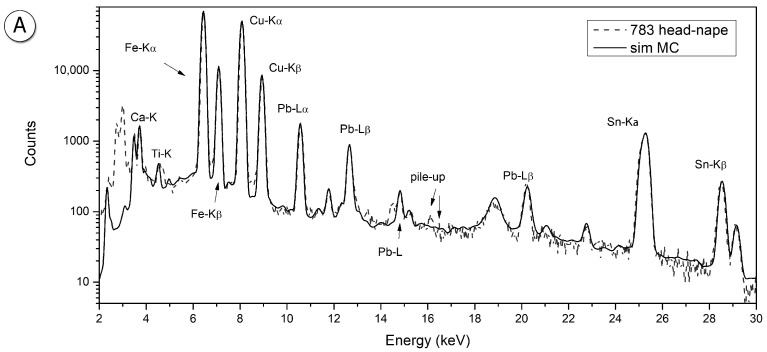

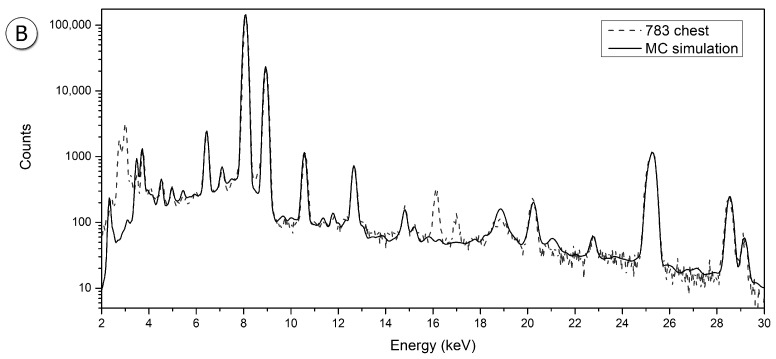
Simulated spectra superimposed to the measured one: sample #783, (**A**) head (**B**) and chest.

The #783 statuette analysis shows a similar result, confirming that the head is an iron-covered bronze, while the body is made of a bronze alloy only, already suggested by a visual inspection. Even in this case, the bronze under the surface of the head and the one composing the body did not show significant differences. The simulated XRF and the measured spectra of the head and chest are reported in Figure 3.

Both patinas from both statuettes present the same set of chemical elements, mainly S, Ca, Ti, and Fe, indeed very common. However, the relative concentrations of these elements vary depending on the position over the body. Further, the corresponding zones between the two statuettes show the same type of variation of concentrations of these chemical elements. For example, both show a higher concentration of Ca at the ankles or of S at the head. This may imply a common burial site.

Regarding the thickness of the layers, the protective layer varies from 40 to 70 μm in thickness, depending on the spot analyzed. While for the bronze patina layer, sample #26065 has an average thickness of 70 μm in the head area and an average thickness of 35 μm in the body region. It is also evident that the presence of Fe has caused deeper corrosion. In sample #783, the patina is more uniform in thickness, about 45 μm. Thus, it is interesting to note that, unlike what was observed at a first glance, the state of conservation of sample #783 seems better than that of #26065. The reduction of the corrosion layer in sample #783 is due to previous cleaning interventions, where, as indicated in the restoration reports, even a mechanical cleaning took place.

## 4. Discussion and Conclusions

Two quite similar statuettes have been investigated with an XRF–MC protocol. Although widely used in previous works, here, its analytical capabilities have been enhanced, allowing us to obtain pieces of information usually not accessible with standard XRF approaches. The two statuettes are almost identical in composition and aspect. This implies their provenience is from one same manufacturing site, even if they are separately preserved in two different museums, and their provenience unknown; although they are both findings from before the 20th century.

However, the most interesting results regard the Fe corrosion on the statues’ heads. Covering exclusively the head precludes the hypothesis of an interaction with a Fe-rich environment, as in this case the corrosion should cover the whole body. Moreover, it is unlikely to be a result of a fusion over the head, due to the lack of technology at the time. In the Sardinian Early Iron Age, the required melting temperature for iron was hardly reached. The only possible explication is the use of a decorative layer, maybe Fe-based paintings, such as ochres. There is no evidence of paintings over bronze objects, only on rocky walls. This aspect must be thoroughly examined, and at least one of the samples must be further investigated with neutron spectroscopic/imaging techniques, which perhaps will give more information. Meanwhile, a systematic search for more statuettes or other Nuragic bronzes with the same kind of Fe-rich layer must be carried out.

## Figures and Tables

**Table 1:** Average alloy composition, expressed in wt%, of the head and body area of samples #26065 and #783.

Inventory Number	Area	Copper (Cu %)	Tin (Sn %)	Lead (Pb %)
26065	Head	86.8	8.3	4.5
	Body	86.5	8.6	4.7
783	Head	91.0	7.1	1.7
	Body	90.6	7.1	2.2

**Table 2:** Iron concentration at different spots.

Sample	Measurement Points	Iron (Fe %)	Graphic Representation
26065	(a) Right side neck	15.0	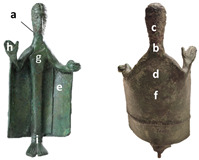
	(b) Neck	19.7	
	(c) Nape	26.3	
	(d) Cloak	0.8	
	(e) Inner cloak on the left	0.7	
	(f) Center back	1.0	
	(g) Chest	0.8	
	(h) Right hand	0.7	
	(i) Ankles	1.3	
783	(1) Head left	20.0	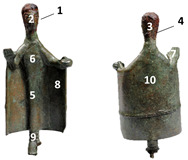
	(2) Nose	22.0	
	(3) Nape	31.3	
	(4) Right side neck	10.5	
	(5) Bust - belt	0.6	
	(6) Chest	0.8	
	(7) Right hand back	0.9	
	(8) Inner cloak on the left	1.0	
	(9) Ankles	1.0	
	(10) Back	0.5	

## Data Availability

The data generated is available upon request to the authors.
